# The Impact of Locoregional Therapy in Nonmetastatic Inflammatory Breast Cancer: A Population-Based Study

**DOI:** 10.1155/2018/6438635

**Published:** 2018-06-03

**Authors:** Mahvish Muzaffar, Helen M. Johnson, Nasreen A. Vohra, Darla Liles, Jan H. Wong

**Affiliations:** ^1^Department of Medicine, Division of Hematology/Oncology, East Carolina University Brody School of Medicine, Greenville, NC, USA; ^2^Department of Surgery, Division of Surgical Oncology, East Carolina University Brody School of Medicine, Greenville, NC, USA

## Abstract

**Background:**

Inflammatory breast cancer (IBC) is a rare but most aggressive breast cancer subtype. The impact of locoregional therapy on survival in IBC is controversial.

**Methods:**

Patients with nonmetastatic IBC between 1988 and 2013 were identified in the Surveillance, Epidemiology, and End Results (SEER) registry.

**Results:**

We identified 7,304 female patients with nonmetastatic inflammatory breast cancer (IBC) who underwent primary tumor surgery. Most patients underwent total mastectomy with only 409 (5.6%) undergoing a partial mastectomy. In addition, 4,559 (62.4%) were also treated with radiation therapy. The patients who underwent mastectomy had better survival compared to partial mastectomy (49% versus 43%, *p* = 0.003). The addition of radiation therapy was also associated with improved 5-year survival (55% versus 40%, *p* < 0.001). Multivariate analysis showed that black race HR (1.22, 95% CI 1.18–1.35), ER negative status (HR 1.22, 95% CI 1.16–1.28), and higher grade (HR 1.14, 95% CI 1.07–1.20) were associated with poor outcome. Cox proportional hazards model showed that total mastectomy (HR 0.75, 95% CI 0.65–0.85) and radiation (HR 0.64, 95% CI 0.61–0.69) were associated with improved survival.

**Conclusions:**

Optimal locoregional therapy for women with nonmetastatic IBC continues to be mastectomy and radiation therapy. These data reinforce the prevailing treatment algorithm for nonmetastatic IBC.

## 1. Introduction

Inflammatory Breast Cancer (IBC) is an uncommon, aggressive breast cancer subtype that accounts for approximately 2% of breast cancers diagnosed annually in the United States [1]. IBC is defined by specific clinicopathologic features including edema (peau d'orange) and erythema of more than one-third of the skin and often has microscopic evidence of dermal lymphatic tumor emboli [2]. IBC is associated with a locoregional recurrence rate of 19.5% [3] and extremely poor long-term outcomes with a median survival of 2.9–3.8 years [4, 5].

Historically, treatment of IBC with surgery and/or radiotherapy alone resulted in five-year survival rates of less than 5% [6]. The use of preoperative chemotherapy has been associated with significant improvements in overall survival. Current treatment guidelines for IBC [7, 8] recommend sequential trimodality therapy consisting of preoperative anthracycline-based polychemotherapy (including targeted therapy depending on HER2 receptor status) followed by total mastectomy with a level I/II axillary node dissection and postmastectomy radiation. Hormonal therapy is prescribed as indicated by the status of the estrogen and progesterone receptors [9].

Locoregional therapy has historically been considered important for the control of symptomatic chest wall disease and/or the prevention of locoregional recurrence. Whether the addition of locoregional therapy to anthracycline-based chemotherapy impacts survival in IBC remains controversial, especially the extent of surgery [10]. Several authors [11, 12] have reported similar survival rates in small cohort studies with patients who underwent breast conserving surgery when compared to patients who underwent total mastectomy after preoperative systemic therapy and have questioned whether IBC is an absolute contraindication to breast conservation therapy. However, these reports are from single institutions and have a small sample size. The purpose of this study is to examine the impact of locoregional therapy, particularly the extent of surgical therapy, on survival in a large population cohort of women with nonmetastatic IBC.

## 2. Materials and Methods

### 2.1. Patient Population

**Figure 1:**
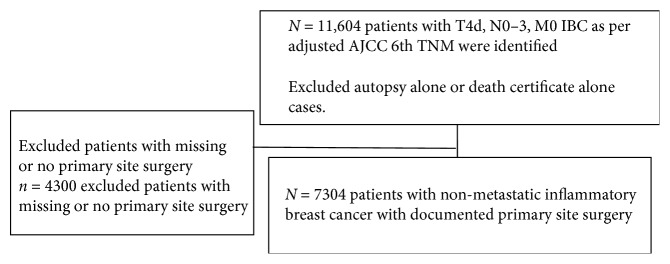


We obtained data from National Cancer Institute's Surveillance, Epidemiology, and End Results (SEER) registries. SEER currently collects and publishes cancer incidence and survival data from population-based cancer registries that cover approximately 28 percent of the US population. A query of the SEER registry using SEER^*∗*^Stat 8.1.2 was performed to identify patients with nonmetastatic IBC diagnosed between the years January 1988 and December 2013. The search was restricted to females with Adjusted AJCC 6 Stage III (T4d, N0–3, M0) IBC. IBC was also defined according to the AJCC sixth edition and was categorized and coded within the SEER registry database as T4d disease. According to the AJCC sixth edition, IBC is defined as being characterized by diffuse erythema and edema (peau d'orange) of the breast, frequently without an underlying palpable mass. We collected demographic data (age at diagnosis, race) and tumor characteristics (stage, histologic subtype, estrogen (ER) and progesterone (PR) receptor expression status, and HER2 overexpression status). For selection of cases, see Figure 1.

Patients with Procedure Codes 20–24 were considered to have had a partial mastectomy, while patients with Procedure Codes 30–80 were considered to have had a mastectomy. Patients with Procedures Codes 00 (no surgery performed), 90 (surgery, NOS), or 99 (unknown if surgery was performed) were excluded from the analysis.

Patients with the estrogen receptor (ER) status or progesterone receptor (PR) status being borderline were considered positive as per the SEER breast cancer subtype algorithm. Patients with HER2 status reported as either borderline or unknown were not utilized to characterize the breast cancer subtype. SEER began collecting HER2 receptor status data for breast cancer cases in 2010. For this study we divided patients into three subtypes. The hormone receptor positive subtype was defined as estrogen receptor and or progesterone receptor positive and HER2 negative, HER2 ositive subtype as estrogen and or progesterone receptor positive or negative with HER2 positive, and triple negative subtypes as estrogen receptor, progesterone receptor, and HER2 negative.

### 2.2. Statistical Analysis

Univariate comparison between groups was performed by means of the Student *t*-test or chi square test where appropriate. Survival was defined as time from date of diagnosis to date of last contact or date of death. Survival was calculated utilizing the method of Kaplan-Meier and significant differences were determined by the log rank test. The Cox proportional hazards regression model was used to determine hazard ratios for overall (OS) and disease specific survival (DSS). All statistical analyses were performed utilizing NCC Statistical Software (version 10, Kaysville, Utah).

## 3. Results

During the study time, 11,604 women were diagnosed with nonmetastatic IBC. Of these women, 7,304 patients had locoregional treatments documented and formed the basis of this analysis. The majority (6,895 patients) underwent total mastectomy while only 409 (5.6%) underwent partial mastectomy. Of patients who underwent a defined surgical procedure, 4,559 (62.4%) were also treated with radiation therapy.

Table 1 summarizes the characteristics of the study population. Women who underwent total mastectomy when compared with those who underwent partial mastectomy were on average slightly older (mean age at diagnosis 60.1 versus 57.1, *p* < 0.001). Nonwhites comprised a greater percentage of women who underwent partial mastectomy (*p* < 0.001). The partial mastectomy and total mastectomy subgroups had similar rates of ER receptor positivity (*p* = 0.02), PR receptor positivity (*p* = 0.14), and histologic subtype (*p* = 0.36).

**Figure 2:**
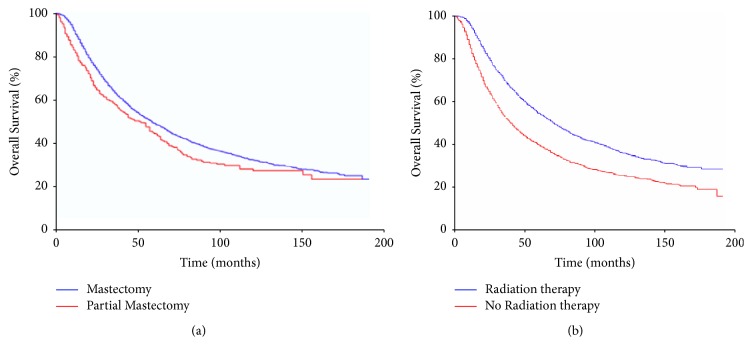
Kaplan-Meier survival curves for patients stratified by type of surgery ((a), *p* = 0.003) and treatment with radiation ((b), *p* < 0.001).

The patients with lower nodal burden, later years of diagnosis, and mastectomy were associated with better outcome (Table 2). Triple negative breast cancer was associated with inferior DSS (HR: 2.40, 95% CI 1.76–3.51), and HER 2-positive group was associated with superior DSS (HR: 0.66, 95% CI 0.44–0.98) but this group had smaller sample size. Age ≥ 50, black race, greater nodal disease burden, ER-negative tumors, PR-negative tumors, and treatment prior to 2000 were associated with inferior outcome (Table 2). Patients who had a mastectomy had a 5-year survival of 49% (median survival 59 mos) compared to a 5-year survival of 43% (median survival 47 mos) in women who had a partial mastectomy (*p* = 0.003) (Figure 2(a)). The addition of radiation therapy was significantly associated with improved overall survival (5-year survival 55% versus 40%, *p* < 0.001) (Figure 2(b)). By multivariate analysis using the Cox proportional hazards model, total mastectomy (HR 0.75, 95% CI 0.65–0.85) and the delivery of radiation (HR 0.64, 95% CI 0.60–0.68) remained significantly associated with improved survival.

## 4. Discussion

Despite substantial improvements in the outcomes of women with IBC over the last several decades, the majority of patients develop and subsequently succumb to metastatic disease [2]. In view of the grave prognosis of IBC, some have questioned whether routine treatment with total mastectomy and level I/II axillary node dissection is necessary for a better outcome given the systemic nature of the disease. In this population-based study of women with nonmetastatic IBC, we sought to examine the impact of locoregional therapy on survival. Our results indicate that total mastectomy compared with partial mastectomy is associated with improved overall and disease specific survival, and the addition of radiation therapy significantly improves overall survival. Although the partial mastectomy and total mastectomy subgroups had several significant demographic differences, partial mastectomy remained independently associated with poorer survival in a Cox regression model. It is possible that the extent of axillary surgery is a confounding variable despite the use of multivariate analysis. The mastectomy subgroup had a greater burden of nodal disease and it has been demonstrated that aggressive locoregional therapy may be more beneficial in patients with high tumor burden [13]. On the other hand, women in the partial mastectomy subgroup were significantly older and more likely to be nonwhite, characteristics frequently associated with worse outcomes [1]. Another possible explanation for poorer outcomes with partial mastectomy is the self-seeding hypothesis, the observation that primary tumors may seed distant sites as well as reseed the primary tumor site [14].

The optimal extent of surgery in IBC is unclear and since earlier studies opted for mastectomy which demonstrated improved survival, mastectomy became an integral part of trimodality therapy in IBC. Recently breast conservation surgery has generated interest due to modernization of both imaging and systemic therapeutic options. Our finding that partial mastectomy is associated with poorer survival contrasts with two small reports on breast conservation therapy in women with IBC. Bonev and colleagues [11] retrospectively analyzed a series of 24 patients with IBC and found no significant difference in overall survival at median follow-up of 60 months between women who underwent partial mastectomy versus total mastectomy following neoadjuvant chemotherapy (overall survival 59% versus 57%, 0.49). Similarly, in a retrospective single-institution analysis, Brzezinska and colleagues [12] found that 35 women with IBC treated with breast conservation surgery had similar rates of locoregional control and survival compared with those treated with mastectomy. The contrast between our finding and the conclusions of these two reports could be explained by differences in patient populations, tumor biology, and variations in treatment regimens. For example, nearly half of the patients in the UK study [12] were treated with neoadjuvant endocrine therapy rather than chemotherapy suggesting predominance of favorable breast subtype in this cohort. In noninflammatory breast cancer, survival following breast conservation therapy has repeatedly been demonstrated to be equivalent to that following mastectomy. The rationale for these trials stemmed from the hypothesis that breast cancer is a systemic disease [15]. Our observation that partial mastectomy is associated with poorer survival for patients may also point towards that IBC is a heterogeneous group of tumors with variable clinical course [16]. Although partial mastectomy was associated with poorer survival in this large, unselected patient population, it is possible that there are subsets of women with IBC for whom breast conservation therapy may be an acceptable alternative to modified radical mastectomy especially for patients with complete clinical and imaging response to preoperative systemic therapy [3].

Genomic profiling, functional imaging, and characterization of additional tumor markers like EXH2 expression [17, 18] may facilitate the identification of tumors for which breast conservation may achieve optimal locoregional control and disease-free survival in future.

Our data also demonstrates that the inclusion of radiation therapy in the multimodal treatment of nonmetastatic IBC impacts survival favorably. This data should be interpreted with caution as SEER does not capture the dose and site specific details of radiation therapy delivered. This is concordant with extant data on noninflammatory breast cancer [19]. Response guided treatment may improve patient selection for more aggressive locoregional treatment in IBC. Patients with suboptimal or nonresponsive disease to preoperative systemic therapy may benefit from preoperative radiation with or without concurrent chemotherapy with some reports in locally advanced breast cancer demonstrating improved response rates and local control [20].

Our data has several limitations inherent to population-based studies. These include missing data as well as lack of treatment details about the systemic therapy, the sequencing of therapies, radiation dosage, and fields of treatment. SEER database does not provide information about the scope of regional lymph node surgery and surgical margin status. While a prospective, randomized controlled trial would be ideal to assess the impact of locoregional therapies on survival, this is a challenge given the rarity and lethality of IBC.

## 5. Conclusions

This study demonstrates that optimal locoregional therapy for women with nonmetastatic IBC consists of both total mastectomy and radiation, not only for control of chest wall disease but also for survival. Further studies are needed to determine whether there may be specific subsets of patients for whom breast conservation therapy may be a reasonable alternative to total mastectomy.

## Figures and Tables

**Table 1:** Study population characteristics.

Variable	Total	Partial mastectomy	Total mastectomy	*p* value
*Number of patients*	7304	409	6895	
*Age (yrs)*				
Mean	56	60.1	57.1	<0.001
Range	21–103	25–97	21–103	
*Race (%)*				<0.001
White	5871 (80.4)	309 (75.6)	5562 (80.7)	
Black	1003 (13.7)	81 (19.8)	992 (13.4)	
Other	430 (5.9)	19 (4.6)	411 (5.9)	
*N stage*				<0.001
N0	1147 (15.7)	147 (35.9)	1000 (14.5)	
N1	2478 (33.9)	143 (35.0)	2335 (33.9)	
N2	1788 (24.5)	66 (16.1)	1722 (25.0)	
N3	1891 (25.9)	53 (13.0)	1838 (26.6)	
*ER status*				0.02
Positive	3458 (47.3)	189 (46.2)	3269 (47.4)	
Negative	3147 (43.1)	165 (40.3)	2982 (43.3)	
Unknown	699 (9.6)	55 (13.5)	644 (9.3)	
*PR* *Status*				0.14
Positive	2671 (36.6)	151 (36.9)	2520 (36.6)	
Negative	3870 (53.0)	204 (49.9)	3666 (53.2)	
Unknown	709 (10.4)	54 (13.2)	763 (10.34)	
*Subtype*				0.36
HR +	579 (38.4)	29 (42.0)	550 (38.2)	
HER2 +	535 (35.5)	19 (27.5)	535 (35.8)	
Triple negative	374 (26.1)	21 (30.5)	395 (25.0)	

**Table 2:** Cox proportional hazards model for overall survival and disease specific survival.

Variable	Overall survival			Disease specific survival		
	*p* Value	HR	95% CI	*p* Value	HR	95% CI
*Node status*	<0.001			*<0.001*		
N0		Referent			*Referent*	
N1		1.26	1.13–1.39		*1.50*	*1.31–1.73*
N2		1.65	1.47–1.83		*2.01*	*1.74–2.31*
N3		2.28	2.05–2.53		*2.90*	*2.50–3.30*
*Year Dx*	<0.001			*<0.001*		
1988–1999		Referent			*Referent*	
2000–2013		0.81	0.75–0.86		*0.77*	*0.70–0.83*
*Surgery*	<0.001			*0.004*		
PM		Referent			*Referent*	
TM		0.75	0.66–0.86		*0.79*	*0.67–0.92*
*Race*	<0.001			*<0.001*		
White		Referent			*Referent*	
Black		1.49	1.37–1.63		*1.60*	*1.43–1.75*
Other		0.84	0.73–0.97		*0.84*	*0.70–0.98*
*Radiation*	<0.001			*<0.001*		
No		Referent			*Referent*	
Yes		0.64	0.60–0.69		*0.7*	*0.65–0.80*
*ER status*	<0.001			*<0.001*		
Positive		Referent			*Referent*	
Negative		1.38	1.26–1.50		*1.40*	*1.30–1.60*
Unknown		1.49	1.15–1.93		*1.63*	*1.21–2.20*
*PR status*	<0.001			*<0.001*		
Positive		Referent			*Referent*	
Negative		1.22	1.11–1.34		*1.35*	*1.22–1.51*
Unknown		0.96	0.75–1.25		*0.97*	*0.72–1.30*
*Age (yrs)*	<0.001			*<0.001*		
<50		Referent				
≥50		1.28	1.19–1.37			
